# Trust and COVID-19 vaccine hesitancy in the Dominican Republic: a national cross-sectional household survey, June–October 2021

**DOI:** 10.1136/bmjopen-2023-081523

**Published:** 2024-05-23

**Authors:** Salomé Garnier, Cecilia Then, Michael de St Aubin, Angela Cadavid Restrepo, Helen J Mayfield, Devan Dumas, William Duke, Farah Peña, Adam J Kucharski, Ronald Skewes, Emily Zielinski Gutiérrez, Julia Coyoli, Marie Caroline Etienne, Colleen L Lau, Marietta Vázquez, Eric Nilles

**Affiliations:** 1 EHESP French School of Public Health, Paris, France; 2 Department of Emergency Medicine, Brigham and Women's Hospital, Boston, Massachusetts, USA; 3 Harvard Humanitarian Initiative, Cambridge, Massachusetts, USA; 4 Dirección General de Epidemiología, Santo Domingo, Dominican Republic; 5 School of Public Health, The University of Queensland, Saint Lucia, Queensland, Australia; 6 Universidad Nacional Pedro Henríquez Ureña, Santo Domingo, Dominican Republic; 7 London School of Hygiene & Tropical Medicine, London, UK; 8 Central America Regional Office, Centers for Disease Control and Prevention, Guatemala City, Guatemala; 9 Department of Government, Harvard University Faculty of Arts and Sciences, Cambridge, Massachusetts, USA; 10 Yale School of Medicine, New Haven, Connecticut, USA

**Keywords:** COVID-19, public health, vaccination, behavior

## Abstract

**Abstract:**

**Objective:**

This study investigates the role of trust in shaping COVID-19 vaccine acceptance in the Dominican Republic (DR) during the COVID-19 pandemic.

**Design:**

Cross-sectional household survey.

**Setting:**

Randomly selected households across 134 clusters in the DR, from 30 June 2021 to 12 October 2021.

**Participants:**

5999 participants ≥16 years of age were enrolled.

**Outcome measures:**

COVID-19 vaccine hesitancy (CVH) data were collected from participants ≥16 years of age and analysed as both an ordinal and binary variable.

**Results:**

Overall, CVH was low (5.2% (95% CI 4.6% to 5.8%)), but more common among younger individuals, women and individuals of Mestizo ethnicity. Higher trust in local government, national government, scientists and local doctors (considered official sources) was associated with lower odds of CVH (OR 0.89 (95% CI 0.72 to 0.88), 0.89 (95% CI 0.81 to 0.98), 0.87 (95% CI 0.80 to 0.94) and 0.70 (95% CI 0.62 to 0.80), respectively). Higher trust in religious leaders, social media and traditional media (considered unofficial sources) was associated with higher odds of CVH, with respective ORs of 1.32 (95% CI 1.18 to 1.47), 1.30 (95% CI 1.19 to 1.41) and 1.08 (95% CI 0.97 to 1.22).

**Conclusion:**

We report findings on CVH from a national household survey in the DR and identify overall low rates of CVH but marked heterogeneity by age, gender and ethnicity. Trust in unofficial versus official sources of information is associated with increased CVH. These findings highlight and quantify the importance of trust as a key parameter when considering public health communication strategies.

**STRENGTHS AND LIMITATIONS OF THIS STUDY**

- Our research contributes to a growing body of knowledge on adult vaccine hesitancy (VH) during global pandemics and is among the first studies to describe adult VH in the Dominican Republic.
- A rigorous multistage household sampling method was used to select a substantial study sample that is largely representative of the Dominican population, though our findings may not be generalisable to other settings.
- Eligible individuals who refused to participate may have had lower levels of trust and higher levels of COVID-19 VH (CVH) than study participants.
- Study data were self-reported, and therefore, potentially subject to social desirability bias.
- The scale used to assess CVH was not a validated instrument, due to the pandemic context and constant evolution of vaccine availability.

## Introduction

 In 2019, the WHO classified vaccine hesitancy (VH) as one of the leading threats to global health.^1^ Defined as a delay in the acceptance or refusal of vaccination despite the availability of vaccination services,^2^ this threat has been markedly exacerbated during the COVID-19 pandemic.^3^ By late 2020, several COVID-19 vaccines had been developed, with large vaccine trials demonstrating their efficacy and safety. Yet, large segments of the global population were, and continue to be, reluctant to accept COVID-19 vaccines.^3^ A multicountry survey in late 2020 found that almost one-third of respondents would not receive a potential vaccine, even if demonstrated to be safe and effective.^4^ In Latin America and the Caribbean, populations have traditionally been receptive to childhood vaccination, but a concerning trend of increased VH, particularly towards COVID-19 vaccines, has emerged during the pandemic.^5^

The Strategic Advisory Group of Experts on VH has identified three primary determinants of modern VH: (1) vaccine and vaccination-specific issues, (2) individual and group influences and (3) contextual influences.^2^ Most existing VH studies have focused on the first two categories while the systemic or contextual reasons for VH are rarely addressed and are poorly understood, missing an opportunity to understand contextual influences underpinning VH. Trust beyond healthcare providers, including trust in the local and national government, traditional media sources, and religious leaders, appears to be a critical contextual factor that drives behaviour and adherence to public health guidance during disease outbreaks.^26^,^8^ By extension, trust is a key element when communicating on vaccines and vaccine recommendations, a point of particular relevance given there is a very small but measurable risk of serious adverse outcomes.^9^,^12^ A study covering multiple Latin American countries found that trust in the information source and mutual partisan or religious identity could increase responsiveness to communications about vaccines, in turn improving vaccine uptake.^13^ Yet, despite the global public health consequences of VH, detailed analyses of how trust influences VH are limited, with most relevant studies focusing solely on trust in government or health authorities.

As such, a more nuanced understanding of trust, including trust in different official or unofficial sources, such as social media, is necessary. Using data enumerated from a national multistage household survey, we measured COVID-19 VH (CVH) in the Dominican Republic (DR) and investigated the role of trust as a contextual factor in shaping vaccine acceptance across a broad array of sources.

## Methods

### Setting

The DR is an upper-middle-income Latin American country that shares the island of Hispaniola with Haiti. With almost 11 million residents, it is the second most populous country in the Caribbean.^14^ Before the COVID-19 pandemic, routine childhood vaccine acceptance was high, as reflected in more than 90% coverage for most childhood vaccines in 2018. In the region, the DR is 1 of 18 out of 33 Latin American and Caribbean countries to have reached and maintained 90% coverage of DPT3 vaccines (combined diphtheria, tetanus toxoid and pertussis vaccine), a target indicator for the region and the world.^15^

The first laboratory-confirmed case of SARS-CoV-2 was reported in the DR on 1 March 2020, and strict public health measures commensurate with most countries in the region were implemented.^16^ By 21 August 2021, the study midpoint, 347 637 cumulative cases and 3989 deaths were reported.^17^ A national COVID-19 vaccination campaign was launched in February 2021 and the DR was the first country in the Americas to authorise third doses for high-risk individuals. The government’s vaccine communication campaign (VacúnateRD, ‘Get vaccinated, DR’) promoted COVID-19 vaccination through billboards, social media posts, messaging by popular urban artists and ambulatory vaccination clinics, among others.^18^ By the study midpoint, 52.3% of the population had received at least one dose of a COVID-19 vaccine, 36.2% had received a two-dose primary vaccine series and 5.3% had a third dose.

### Study design, study sites, participant selection

As previously reported, we conducted a three-stage cross-sectional national household serological survey between 30 June 2021 and 12 October 2021.^19^ A total of 134 clusters were selected from 12 565 communities, representing one of every 93 communities in the country. After dividing the country into five regions, we assigned the number of clusters to each of the 31 provinces plus the Santo Domingo National district by proportion of the national population while also considering spatial distribution and urban versus rural environments. Second, we selected clusters by province using a spatially representative sampling method and applied grid methods in urban areas designed to maximise the spatial dispersion of clusters.^20^ A total of 23 households per cluster were selected for enrolment using similar methods. Two provinces, where longitudinal enhanced acute febrile infection surveillance is conducted, were oversampled with 60 households per cluster enrolled. Third, we selected households using satellite images and grid methods.^21^ Household members aged ≥5 years old present in the home at the time of the serological survey were invited to participate, but only those ≥16 were included in the VH component. One head of household ≥18 was enrolled in each household (online supplemental figure S1).

Written consent was obtained from all participants. For children <18 years old, except emancipated minors, consent was obtained from the legal guardian. Written assent was provided by adolescents 16–17 years.

### Study procedures

Trained field research teams were deployed to assigned clusters and sought out selected households using Global Positioning System (GPS) coordinates. Team supervisors introduced the study to household members, verified eligibility and obtained written informed consent prior to data collection. Consent was obtained in the participant’s preferred language (Spanish, Creole or English). If the participant or legal guardian was illiterate, the relevant informed consent/assent forms were verbally read to them by staff in their language of preference, and a thumbprint was provided in lieu of a signature. Questionnaires were verbally administered by enumerators in Spanish, Creole or English to study participants, using the KoboToolbox data collection platform (www.kobotoolbox.org) on electronic tablets.

The study instrument included questions on self-reported demographics, household data, and attitudes and perceptions about COVID-19 vaccines, COVID-19 vaccination experiences, routine childhood vaccinations and trust in institutions (online supplemental table S1). The survey questionnaire was designed with skip logic to limit redundancy, and therefore, not all questions were enumerated for all participants (online supplemental figure S1). Questions about the household were only enumerated for the designated head of household, including trust, usual sources of COVID-19 information and household routine childhood vaccination. Other household members ≥16 were asked about attitudes towards vaccination, but not about trust. Median time to complete the survey was 18 min.

### Data classification and analysis

CVH was analysed both as an ordinal variable (already received a COVID-19 vaccine=0, definitely would accept a vaccine=1, probably would accept a vaccine=2, unsure if they would accept a vaccine=3, probably would not accept a vaccine=4 and definitely would not accept a vaccine=5) and as a binary categorical variable (0=already received, would definitely or would probably accept a COVID-19 vaccine vs 1=unsure, probably would not or definitely would not receive a COVID-19 vaccine). Trust was similarly analysed as both an ordinal variable and a binary categorical variable. Participants were asked separate questions about their level of trust in different actors, including national and local government, scientists, local medical doctors, religious leaders, social media and traditional media (ie, television, radio and newspapers), on a 5-point scale (do not trust at all=1, not very much=2, neutral=3, a little=4, a lot=5). A binary trust variable was created with trust scores of 1–3 considered not trusted and 4 and 5 as trusted.

COVID-19 information sources were aggregated into official vs unofficial information sources. Official sources were defined as those mandated or expected to disseminate information based on available scientific evidence and official guidelines and included local and national government, medical doctors and scientists. Unofficial sources were those without specific mandates to provide evidence-based information and included social media, religious leaders (eg, Catholic priests and Protestant pastors) and traditional media (radio, broadcast television and newspapers).

Trust in official and unofficial sources was assessed using the mean trust score for the respective sources, with mean scores of four or greater considered trustful and less than four as not trustful. Using this binary variable, an individual-level trust profile was constructed wherein each study participant was assigned to one of four categories: trust all, trust-only official, trust-only unofficial and trust none.

Univariable and multivariable logistic regression models were used on the whole sample to assess associations between covariates and CVH as a binary variable, adjusting for clustering at the household level. Specifically, a mixed effects binomial logistic regression model was employed, with household as a random intercept. Proportional odds logistic regressions were then conducted among heads of households to assess associations between CVH as an ordinal variable and trust, as well as between CVH and trust profile. Covariates including age, education level, urban ersus rural household setting, prior reported SARS-Co-V-2 infection, past death of a household member or acquaintance due to suspected or confirmed COVID-19, week in which the respondent was surveyed, province and general degree of trust (calculated as the sum of all trust scores, excluding the measure of trust used as the main independent variable) were included in the models. The latter variable considers whether an individual is generally trustful or not, to mitigate the effect of generally distrustful individuals and better observe the isolated effect of trust in each different actor. Findings were highlighted based on their statistical significance, using a significance level of α=0.05 for hypothesis testing. Additionally, 95% CIs were reported for all estimates. Finally, a heatmap was generated to visualise the relationship between trust and VH, stratified by age group, to highlight potential effect modification by age.

### Patient and public involvement

None.

## Results

Between 30 June 2021 and 12 October 2021, 6741 participants ≥5 years of age out of a total of 7916 eligible individuals (85.2%) present in the household at the time of the survey were enrolled. Of these 5999 were ≥16 years of age and 3899 were enumerated as head of household. More women were enrolled than men (62% vs 37%), and participants were mostly of Mulatto and Mestizo ethnicity (51% and 34%, respectively). Most participants had some primary or secondary education (74%), with a balanced rural/urban and age distribution. Demographic characteristics are detailed in table 1. Heads of households were comparable to study participants in all key variables (online supplemental table S2). Overall, CVH was low with 311/5999 of study participants reporting some degree of CVH (5.2%, 95% CI (4.6% to 5.8%)). Of these, 124/5999 (2.0% (95% CI 1.7% to 2.4%)) reported they would definitely not, 145/5999 (2.4% (95% CI 2.0% to 2.8%)) probably not and 42/5999 (0.7% (95% CI 0.5% to 0.9%)) unsure if they would accept a COVID-19 vaccine at the time of the survey.

**Table 1:** Characteristics of study participants and COVID-19 vaccine hesitancy among participants 16 years of age and older, Dominican Republic, 30 June 2021 to 12 October 2021

Covariate	Participants, n (%)	CVH participants, n	CVH prevalence, % (95% CI)
Overall	5999	311	5.20 (4.64 to 5.78)
Age, years			
16–24	1077 (18)	64	5.94 (4.68 to 7.52)
25–44	1972 (33)	120	6.09 (5.11 to 7.23)
45–64	1880 (32)	90	4.79 (3.91 to 5.85)
≥65	978 (17)	33	3.37 (2.41 to 4.71)
Gender*			
Female	3730 (62)	194	5.20 (4.53 to 5.96)
Male	2227 (37)	115	5.16 (4.32 to 6.16)
Ethnicity†			
Mestizo	2034 (34)	123	6.05 (5.09 to 7.17)
Mulatto	3088 (51)	117	3.79 (3.17 to 4.52)
White	130 (2)	10	7.69 (4.18 to 13.74)
Other	737 (12)	60	8.14 (6.37 to 10.35)
Education			
No education	699 (12)	28	4.01 (2.78 to 5.74)
Some education	4440 (74)	251	5.65 (5.01 to 6.37)
Higher education	841 (14)	30	3.57 (2.50 to 5.06)
Household setting‡			
Rural	2761 (46)	158	5.72 (4.92 to 6.65)
Urban	3238 (54)	153	4.73 (4.05 to 5.51)

Ethnicity and education were self-reported by survey participants. Participants with primary (n=2113) or secondary (n=2327) education were combined into ‘some education’. Participants with technical (n=115) or university (n=726) education were combined into ‘higher education’. Participants noted as having ‘no education’ self-reported as having no formal education. Missing values for education (n=19) and ethnicity (n=10) were omitted from the table.

* Gender includes 42 participants who self-identified as ‘other’ or ‘prefer not to say’, none were vaccine hesitant.

† Mestizo is used to describe people of mixed ancestry with a white European and an indigenous background. Mulatto describes people of African and European descent.

‡ Clusters were defined as rural or urban based on data provided by the Dominican Ministry of Health.

Older age groups reported lower CVH when compared with the 25–44 year reference group, particularly those 65 years of age and older, with a similar but non-statistically significant trend among the 45–64 years age group (table 2). Men reported statistically significantly lower CVH than women. Individuals who self-identified as Mulatto ethnicity were less likely to be VH than individuals self-identifying as Mestizo. Those with higher education were less likely to be VH than those with some formal education (ie, primary or secondary education) in the univariate model, though the association was not statistically significant in the multivariate model.

**Table 2:** Univariable and multivariable predictors of COVID-19 vaccine hesitancy among participants 16 years of age and older, Dominican Republic, 30 June 2021 to 12 October 2021

Variable	Univariable ORs (95% CI)	P value	Multivariable ORs (95% CI)	P value
Age, years				
16–24	0.97 (0.71 to 1.33)	0.871	1.13 (0.48 to 2.68)	0.774
25–44*	Ref		Ref	
45–64	0.77 (0.58 to 1.02)	0.066	0.83 (0.36 to 1.88)	0.648
≥65	0.55 (0.37 to 0.80)	0.002	0.19 (0.06 to 0.61)	0.005
Gender				
Female	Ref		Ref	
Male	0.98 (0.77 to 1.24)	0.883	0.51 (0.27 to 0.98)	0.043
Ethnicity				
Mestizo	Ref		Ref	
Mulatto	0.61 (0.47 to 0.79)	<0.001	0.36 (0.15 to 0.89)	0.027
White	1.29 (0.62 to 2.41)	0.45	1.74 (0.21 to 14.48)	0.611
Other	1.38 (0.99 to 1.89)	0.051	1.24 (0.34 to 4.54)	0.747
Education				
No formal education	0.70 (0.46 to 1.02)	0.077	1.15 (0.35 to 3.83)	0.814
Some education*	Ref		Ref	
Higher education	0.62 (0.41 to 0.89)	0.015	0.67 (0.21 to 2.13)	0.495
Household setting				
Rural	Ref		Ref	
Urban	0.82 (0.65 to 1.04)	0.096	0.81 (0.03 to 18.93)	0.897

Ethnicity was self-reported by survey participants. Participants with primary (n=2113) or secondary (n=2327) education were combined into ‘some education’. Participants with technical (n=115) or university (n=726) education were combined into ‘higher education’. All variables in the table were included in the multivariate model, which was adjusted for clustering at the household level. N=5989.

* Included most participants, and therefore, chosen as a reference value.

Refreference

### Trust and CVH

Higher trust in local government, national government, scientists and local doctors was independently associated with lower odds of CVH. ORs for these actors were 0.89 (95% CI 0.72 to 0.88, p<0.001), 0.89 ((95% CI 0.81 to 0.98, p<0.017), 0.87 ((95% CI 0.80 to 0.94, p<0.001) and 0.70 ((95% CI 0.62 to 0.80, p<0.001), respectively (online supplemental table S3). Conversely, higher trust in religious leaders, social media and traditional media was associated with higher odds of CVH, with respective ORs of 1.32 ((95% CI 1.18 to 1.47, p<0.001), 1.30 ((95% CI 1.19 to 1.41, p<0.001) and 1.08 ((95% CI 0.97 to 1.22, p=0.2). When analysing trust profiles and considering trust-only official sources as the reference category, trust-only unofficial, trust all and trust none profiles were each associated with increased odds of CVH by 2.16 ((95% CI 1.38 o 3.40), 2.49 ((95% CI 1.90 to 3.27) and 3.36 ((95% CI 2.42 to 4.65), respectively (p<0.001, online supplemental table S4).

**Figure 1:**
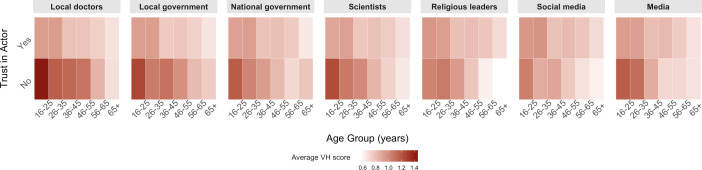
COVID-19 vaccine hesitancy (VH) score by age, trust and actor among heads of households, Dominican Republic, 30 June 2021 to 12 October 2021. Darker shading represents higher levels of VH. Trust is presented here as a binary variable with trusting ‘a lot’ and ‘a little’ as ‘yes’ and other categories as ‘no’. VH is presented as the average VH score for individuals in each category, from 0 (already vaccinated) to 5 (will definitely not receive a vaccine in the future).

When considering CVH stratified by age and trust in various sources of COVID-19 information, we observed that CVH was highest among young individuals with low trust (figure 1) and among participants who distrust local doctors, local government and national government. Generally, CVH was higher among distrustful individuals than among trustful individuals, except among older people who distrust religious leaders and social media, among whom CVH is lowest.

## Discussion

We reported findings on CVH from a national household survey in the DR and identified overall low rates of CVH but marked demographic heterogeneity. Multiple demographic variables, including age, ethnicity, gender and education level, were associated with CVH. Trust in unofficial versus official sources of information was associated with a 2.5-fold increase in CVH. These findings highlight and quantify the importance of trust as a key parameter when considering public health communication strategies.

First, older age, Mulatto ethnicity and male gender were associated with lower CVH in multivariate analysis. Higher education was associated with lower CVH only in univariate analysis. Age has been widely reported as important for VH, with overall lower VH among older adults and higher VH among adolescents and young adults.^7 21 22^ Similarly, higher levels of education, particularly tertiary education, are largely associated with lower VH in many settings.^7 22^ Female gender has been identified as a predictor of VH in Latin America, specifically in the COVID-19 context.^23^ This gender difference has been hypothesised to result from higher exposure among women to vaccine misinformation (eg, in social media or mother communities), a higher tendency to believe conspiracy theories, as well as lower perceived vulnerability towards COVID-19.^23^,^25^ Our results do not point to any strong association between VH and residential settings (urban vs rural), which similar studies have observed in the Latin American setting.^23 26^ While most existing studies find notable differences in VH by various demographic factors, there is a consensus that these are context-specific and not as relevant to understanding VH as are personal history and experiences, engagement with health services, tendencies towards conspiratorial beliefs and community influences.^27 28^

Our findings on trust, however, are relevant beyond the DR and the COVID-19 pandemic, as we emphasise the role of contextual and attitudinal factors on health behaviours and acceptance of public health measures. We characterise and quantify the role of trust in a range of institutions and COVID-19 information sources in shaping attitudes towards vaccination, and find that trust in local doctors, scientists and local/national government is associated with lower odds of CVH. On the other hand, trust in religious leaders and social media is associated with significantly higher odds of CVH. In particular, distrust in both official and unofficial actors is associated with the highest odds of CVH. Conversely, distrust in social media and religious leaders among individuals who trust official actors is associated with the lowest odds of CVH.

Our conclusions on trust and CVH are consistent with existing studies, which have generally acknowledged that distrust of the government and of healthcare providers is a significant predictor of antivaccination beliefs and behaviours.^1129^,^31^ A recent experimental study in Latin America found that willingness to receive a COVID-19 vaccine was increased when vaccine endorsement came from a national medical association, compared with messaging from politicians.^13^ The study found actors with less professional medical knowledge to be least effective in communicating about the COVID-19 vaccine. Our study adds nuance to these findings by exploring how the potential effectiveness of communication from non-medical trusted sources depends largely on individual factors, including age and level of trust in unofficial versus official actors. Besides this recent study, which begins to grapple with the role of media and religious leaders in communicating about the vaccine, most existing research fails to explore trust beyond health and governmental actors. Increasing attention has been placed on community as a determinant of vaccine uptake,^32 33^ but the role of trust in opinion leaders within a given community has yet to be explored in depth.

In the DR and elsewhere, religious leaders such as priests and pastors are broadly perceived as authoritative figures in spiritual and secular matters, which can include health-related decisions. During the COVID-19 pandemic, Dominican research partners expressed concerns about the role of religious leaders in spreading misinformation about the COVID-19 vaccine, with some associating it with an instrument of Devil control. The role of these leaders in influencing VH may differ across denominations, reflecting their respective historical relationships with the state, the presence of a centralised doctrinal authority, and their stances on medical interventions. Further research on the differential role of religious leaders by religious denominations may yield useful insights into the mechanisms linking religion to VH.

Understanding the ways in which trust affects VH is crucial to supporting public health strategies during emergencies beyond COVID-19. Throughout the COVID-19 pandemic, unvaccinated people were over-represented in hospitalisations and deaths.^33^ Their overwhelming presence in hospitals had direct consequences on the availability of healthcare and increased the financial burden on health systems.^33 34^ Vaccine uptake is an example of a public health measure that provides high returns at a population level while the individual benefits are not always obvious nor always outweigh individual costs. Recent COVID-19 literature points to the importance of trust for compliance with other costly public health policies, including compliance with stringent lockdowns, mobility reductions, social distancing and practising hand hygiene.^35^,^37^ As trust is becoming a crucial determinant of adherence to public health measures, understanding which actors are relevant to health-related decision-making, beyond health and political authorities and by age group, can yield important returns at a population level.

This study has multiple strengths. We used a rigorous multistage household sampling method to select study participants from all 32 provinces and used detailed survey tools and univariable and multivariable models to better characterise the role of trust in CVH. Our study sample is large and largely representative of the Dominican population. In addition, it is the first study to describe adult VH in the DR, and one of few studies to explore VH outside of Europe, the USA, and largely populated countries. Finally, our research contributes to a growing body of knowledge on adult VH during global pandemics, which differs significantly from VH towards routine childhood vaccination.

Still, there are several limitations to our study. First, household members who refused to participate may have had different perceptions of trust and vaccination than those enrolled. Specifically, we anticipate that non-participants may have lower levels of trust in established institutions, and therefore, higher levels of CVH than study participants. Additionally, study data were self-reported, and therefore, potentially subject to social desirability bias. The scale used to assess CVH was not a validated instrument, mostly because of the pandemic context and constant evolution of vaccine availability. Finally, the study was conducted in a single country, and the findings may not be generalisable to other settings. Adjustment for complex study design was not performed, given that analysis focused on identifying risk factors rather than prevalence.

Our study shows that building trust is crucial for vaccine uptake. Yet, maintaining trust can be challenging during a national crisis. From a public health perspective, it is essential to understand how best to encourage such socially desirable behaviours, not necessarily by making them mandatory, but rather by building trust in the entities that promote them. This study addresses conditions that influence people to adhere to public health policies, suggesting that trust in authorities and experts remains crucial for effective governance and citizen cooperation. Our findings suggest that leveraging trusted unofficial actors in official public health communications could be key to building trust in public health recommendations. Future research on the issue should focus on how to build and maintain trust in official actors, especially in a context of crisis. Understanding trust as a multidimensional concept and exploring the factors that shape its occurrence and strength will be crucial to developing trust-building policies.

## supplementary material

10.1136/bmjopen-2023-081523 online supplemental file 1

## Data Availability

Data are available on reasonable request.
