# Supplementary material for: Trust and COVID-19 vaccine hesitancy in the Dominican Republic: a national cross-sectional household survey, June–October 2021
Source: BMJ Open. 2024 May 23;14(5):e081523. doi: 10.1136/bmjopen-2023-081523 (PMC11328667; doi:10.1136/bmjopen-2023-081523)
Supplement: online supplemental file 1 [file bmjopen-14-5-s001.pdf]

SUPPLEMENTAL MATERIAL

Figure S1: Overview of survey sample breakdown and corresponding survey topics.

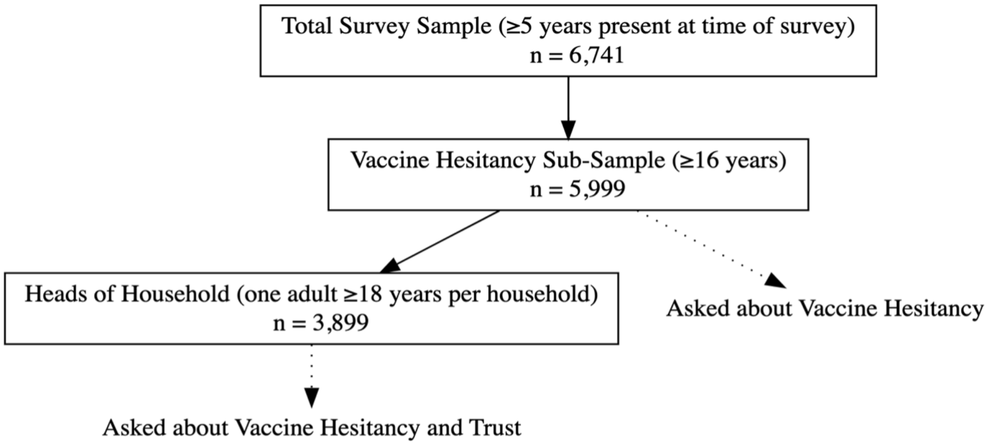

Table S1: Relevant questions from the survey instrument.

1. **Demographic Information** *(Asked to all participants)*	
**C.10. Date of Birth**	
**C.17. Gender**	❑ Male<br>❑ Female<br>❑ Other<br>❑ Prefer not to say
**C.28. Ethnicity**<br>(Patient Reported)	❑ Caribbean-European (Mulatto)<br>❑ African-Caribbean (Mestizo)<br>❑ Indigenous<br>❑ White<br>❑ Other<br>❑ Don't know/Prefer not to say
**C.30. What is the highest level of school the person has completed?**	❑ No Formal Schooling Primary (1-8)<br>❑ Secondary (9-12)<br>❑ Technical/Vocational School<br>❑ University/Tertiary<br>❑ Don't know/Prefer not to say
**E.1. Is the house in an informal settlement (*barrio*)?**	❑ Yes<br>❑ No<br>❑ Don't know/Prefer not to say
2. **Vaccination History** *(Asked only to the head of household)*	
**D.10. Have your children received all of the recommended routine childhood vaccines?**	❑ Yes, all my children have had all of the vaccines<br>❑ Yes, some of my children or some of the vaccines<br>❑ No, none of my children have had any of the vaccines<br>❑ I don't know
**D.11. If no/if some, why?**	❑ Did not think they were needed<br>❑ Did not think the vaccines were effective<br>❑ Did not think the vaccines were safe/ concerned about side effects<br>❑ Had a bad experience or reaction with previous vaccination<br>❑ Religious reasons<br>❑ Other beliefs/traditional medicine<br>❑ Fear of needles<br>❑ Not possible to leave work (at home or other)<br>❑ Cost-related reasons<br>❑ Other (explain)
**D.12. If other, specify**	

**3. Information and Trust** *(Asked only to the head of household)*	
**F.1. In the last 12 months, from which of the following sources has the person received health information? This includes but is not limited to information about COVID-19.** (check all that apply)	❑ School<br>❑ WhatsApp<br>❑ Facebook<br>❑ Twitter<br>❑ Instagram<br>❑ Snapchat<br>❑ Brochures<br>❑ Internet (non-social media)<br>❑ Newspapers<br>❑ Radio<br>❑ Health Professionals<br>❑ TV<br>❑ Neighbors<br>❑ Family/Friends<br>❑ Co-workers<br>❑ Relatives in the United States<br>❑ Government Officials<br>❑ None<br>❑ Other
**F.3. In general, to what extent do you trust the following actors?**<br>*1 = Not at all, 2 = Not very much, 3 = Neutral, 4 = A little, 5 = A lot*	❑ Local doctor/local health center<br>❑ Local government<br>❑ Local religious leaders<br>❑ The media (radio, TV, newspapers)<br>❑ Social media (WhatsApp, Facebook, Instagram, Twitter, etc)<br>❑ National government<br>❑ Scientists
**4. COVID-19 and Vaccine** *(Asked to all participants over 16)*	
**G.23. How likely is it that you previously had COVID?**	❑ Certain<br>❑ Very Likely<br>❑ Likely<br>❑ Unlikely<br>❑ Very unlikely<br>❑ Don't know
**G.24. Did anyone in your household die of COVID?**	❑ Yes<br>❑ No<br>❑ Don't know<br>❑ Prefer not to say
**G.25. Do you personally know anyone outside your household who died of COVID?**	❑ Yes<br>❑ No<br>❑ Don't know/Prefer not to say
**G.26. If it were available to you right now, would you accept a COVID-19 vaccine?**	❑ Yes, definitely<br>❑ Probably<br>❑ Probably not<br>❑ No, definitely not<br>❑ Don't know/Not sure<br>❑ Already vaccinated
**G.29. If no/probably not/not sure, why not?**	❑ Do not think it is needed<br>❑ Do not think the vaccine is effective<br>❑ Do not think the vaccine is safe/concerned about side effects<br>❑ Vaccine development was rushed/vaccine not ready<br>❑ Fear of government tracking/surveillance<br>❑ Wants to wait for others to be vaccinated first<br>❑ Had a bad experience or reaction with previous vaccination<br>❑ Religious reasons<br>❑ Other beliefs/traditional medicine<br>❑ Fear of needles<br>❑ Not possible to leave work (at home or other)<br>❑ Cost-related reasons<br>❑ Other (explain)
**G.31. Do you think you will accept a COVID-19 vaccine in the future?** i.e., in several months, or after most of the population has been vaccinated	❑ Yes<br>❑ Probably<br>❑ Probably not<br>❑ No<br>❑ Don't know/Not sure

Table S2: Characteristics of study participants ≥16 and heads of households, Dominican Republic, June 30 to October 12, 2021.

Covariate	Participants, n (%)	Household representatives, n (%)
Overall	5,999	3,899
Age group, years		
16–24	1,077 (18%)	424 (11%)
25–44	1,972 (33%)	1,344 (34%)
45–64	1,880 (32%)	1,399 (36%)
65+	978 (17%)	732 (19%)
Ethnicity		
Mestizo	2,034 (34%)	1,273 (33%)
Mulatto	3,088 (51%)	1,990 (51%)
White	130 (2.2%)	101 (2.6%)
Other	737 (12.2%)	527 (14%)
Education Group		
No education	699 (12%)	441 (11%)
Some education	4,440 (74%)	2,891 (74%)
Higher education	841 (14%)	553 (14%)
Gender		
Female	3,730 (62%)	2,548 (65%)
Male	2,227 (37%)	1,322 (34%)
Urban		
Rural	2,761 (46%)	1,800 (46%)
Urban	3,238 (54%)	2,099 (54%)

Table S3: Proportional odds logistic regression results for COVID-19 vaccine hesitancy and trust among heads of households, Dominican Republic, June 30 to October 12, 2021.

	Vaccine Hesitancy OR (95% CI)						
	(1) Local Doctor	(2) Local Government	(3) National Government	(4) Scientists	(5) Religious Leaders	(6) Social Media	(7) Media
Local Doctor	**0.70*** (0.62, 0.80)**						
Local Government		**0.80*** (0.72, 0.88)**					
National Government			**0.89* (0.81, 0.98)**				
Scientists				**0.87*** (0.80, 0.94)**			
Religious Leaders					**1.32*** (1.18, 1.47)**		
Social Media						**1.30*** (1.19, 1.41)**	
Media							1.08 (0.97, 1.22)
Age	0.99*** (0.98, 0.99)	0.99*** (0.98, 0.99)	0.99*** (0.98, 0.99)	0.99*** (0.98, 0.99)	0.99*** (0.98, 0.99)	0.99*** (0.98, 0.99)	0.99*** (0.98, 0.99)
Higher Education	**Ref**	**Ref**	**Ref**	**Ref**	**Ref**	**Ref**	**Ref**
No Formal Education	2.02*** (1.55, 2.63)	2.1*** (1.62, 2.74)	2.08*** (1.60, 2.71)	2.1*** (1.61, 2.73)	2.08*** (1.59, 2.70)	2.13*** (1.64, 2.78)	2.07*** (1.59, 2.70)
Some Education	1.36*** (1.13, 1.64)	1.39*** (1.15, 1.68)	1.39*** (1.15, 1.67)	1.39*** (1.15, 1.68)	1.4*** (1.16, 1.69)	1.37*** (1.14, 1.66)	1.38*** (1.15, 1.67)
Rural	**Ref**	**Ref**	**Ref**	**Ref**	**Ref**	**Ref**	**Ref**
Urban	0.78*** (0.67, 0.90)	0.78*** (0.68, 0.90)	0.78*** (0.68, 0.90)	0.78*** (0.68, 0.90)	0.78*** (0.68, 0.90)	0.76*** (0.66, 0.88)	0.79*** (0.68, 0.91)
Trustfulness	1.02 (1.00, 1.04)	1.01 (0.99, 1.03)	1 (0.98, 1.02)	1 (0.98, 1.02)	0.93*** (0.92, 0.95)	0.93*** (0.91, 0.95)	0.96*** (0.94, 0.99)
COVID-19 Experience	1.31*** (1.15, 1.49)	1.33*** (1.17, 1.51)	1.33*** (1.17, 1.52)	1.35*** (1.19, 1.54)	1.32*** (1.16, 1.50)	1.34*** (1.18, 1.53)	1.34*** (1.18, 1.52)
Observations	3840	3840	3840	3840	3840	3840	3840
RMSE	1.27	1.27	1.27	1.27	1.27	1.27	1.27
AIC	8363.1	8372.1	8386	8380.2	8359.9	8344.9	8386.9

$^{*}p < 0.05$; $^{**}p < 0.01$; $^{***}p < 0.001$

Proportional odds ratios for COVID-19 vaccine hesitancy on trust, controlling for age, urban/rural, education, COVID-19 experience, and trustfulness. Fixed effects for week and province included in the model but omitted from the table. Each measure of trust has its own regression to account for potential confounding. Confidence intervals included in parentheses.

Table S4: Proportional odds logistic regression results for COVID-19 vaccine hesitancy and trust profile among heads of households, Dominican Republic, June 30 to October 12, 2021.

	Odds Ratio	95% CI	p-value
Trust only official	**Ref**	**Ref**	**Ref**
Trust only unofficial	2.16	1.38, 3.40	<0.001
Trust all	2.49	1.90, 3.27	<0.001
Trust none	3.36	2.42, 4.65	<0.001
Age	0.99	0.98, 0.99	<0.001
Higher education	**Ref**	**Ref**	**Ref**
Some education	1.42	1.17, 1.74	<0.001
No formal education	2.13	1.64, 2.77	<0.001
Rural	**Ref**	**Ref**	**Ref**
Urban	0.82	0.71, 0.94	0.005
Observations: 3885			
AIC: 8472			
RMSE: 1.28			

Proportional odds ratios for COVID-19 vaccine hesitancy and trust profile, controlling for age, rural/urban, and education. Fixed effects for week and province included in the model but omitted from the table.
