# Supplementary material for: Trust and COVID-19 vaccine hesitancy in the Dominican Republic: a national cross-sectional household survey, June–October 2021
Source: BMJ Open. 2024 May 23;14(5):e081523. doi: 10.1136/bmjopen-2023-081523 (PMC11328667; doi:10.1136/bmjopen-2023-081523)
Supplement: Supplementary file 2 [file bmjopen-2023-081523.reviewer_comments.pdf]

PEER REVIEW HISTORY

BMJ Open publishes all reviews undertaken for accepted manuscripts. Reviewers are asked to complete a checklist review form (**http://bmjopen.bmj.com/site/about/resources/checklist.pdf**) and are provided with free text boxes to elaborate on their assessment. These free text comments are reproduced below.

This paper was submitted to a another journal from BMJ but declined for publication following peer review. The authors addressed the reviewers' comments and submitted the revised paper to BMJ Open. The paper was subsequently accepted for publication at BMJ Open.

ARTICLE DETAILS

**TITLE (PROVISIONAL)**	Trust and COVID-19 vaccine hesitancy in the Dominican Republic: a national cross-sectional household survey, June–October 2021
**AUTHORS**	Garnier, Salomé; Then, Cecilia; de St Aubin, Michael; Cadavid Restrepo, Angela; Mayfield, Helen; Dumas, Devan; Duke, William; Peña, Farah; Kucharski, Adam; Skewes, Ronald; Zielinski Gutiérrez, Emily; Coyoli, Julia; Etienne, Marie Caroline; Lau, Colleen L.; Vázquez, Marietta; Nilles, Eric

VERSION 1 – REVIEW

**REVIEWER**	O'Malley, Patricia<br>Premier Health System Support- Nursing Research, Nursing Research
**REVIEW RETURNED**	12-Mar-2024

**GENERAL COMMENTS**	Overall this is a well written report. Statistical analysis is so well done that identifies the relationships (if any) between variables of interest.<br><br>The only weaknesses I identified are:<br>1. In the study procedures section, this information concerning methods is weak (consent, methods for survey delivery to subjects, average time to complete the survey, what questions were used to assess attitude and practices). Was the survey read to those subjects (if any unable to read?). Who accomplished surveys of the large number of subjects? Were other individuals trained to deploy/record etc? How was the survey invitations delivered to subjects. I think the reader would like to see the survey questions.<br><br>2. The use of the term religious leaders is very broad. How would persons in DR perceive or define what religious leaders mean? This is important since this role was associated with VH- and can the authors hypothesize why? I would think knowing who are the "religious leaders" are would be important for providing implications for decreasing VH.<br><br>Also, a bit of discussion around gender differences in the results would be very interesting.

**REVIEWER**	Tarr, Philip

	University of Basel, Universty Dept of Medicine
**REVIEW RETURNED**	17-Mar-2024

**GENERAL COMMENTS**	Interesting cross-sectional quantitative survey looking into covid-19 vaccine hesitancy (VH) in June-October 2021, after/when covid-19 vaccines became locally available, among a representative sample of household members in the Dominican Republic.<br><br>Authors did not apply an established VH instrument, but simply asked participants about intention to obtain covid-19 vaccine. In addition, investigators asked individual, single questions about trust in official (doctors, authorities) and inofficial (social media, religious leaders, traditional media) vaccine information sources.<br><br>Overall, covid-19 VH was low (around 5% of the sample), and predictors of VH, even with substantial relative impact (odds ratios >2), had a limited absolute impact on VH.<br><br>Authors correctly point out as limitation that people with higher levels of VH may well have declined study participation.<br><br>VH towards covid-19 vaccine that was captured in 2021 has limited relevance to today's issues (e.g. has VH increased during the pandemic ? has the pandemic led to increased VH towards established childhood vaccines?)<br><br>Also, findings on trust and VH are consistent with previous reports, but the present report is nonetheless welcome because it provides context-specific information related to the Dominican Republic, because of the large sample size and aim to obtain a representative sample.<br><br>Specific comments:<br><br>- Figure 1: consider relabeling title and legend as "Vaccine hesitancy SCORE" instead of simply "vaccine hesitancy", because readers may well confuse VH prevalence (in percent) with VH score<br><br>- In the MS title, authors correctly make clear that their study is about VH in relation to covid-19 vaccines, however, this should be made clear throughout the text, i.e. replace all "VH" statements by "covid-19 VH", because study is only about covid-19 vaccine and not about HPV vaccine, pneumococcal, meningococcal, influenza, childhood vaccines etc. In particular, this applies to:<br>o abstract methods, results, conclusion<br>o intro (line 33)<br>o methods (section data classification and analysis)<br>o results (line 14)<br>o titles of all tables and figures, including tables S2 and S3

VERSION 1 – AUTHOR RESPONSE

Reviewer: 1
Dr. Patricia O'Malley, Premier Health System Support- Nursing Research, Indiana University East
Comments to the Author:

Overall this is a well written report. Statistical analysis is so well done that identifies the relationships (if any) between variables of interest.

Author response: Thank you for your time and thoughtful comments.

The only weaknesses I identified are:
1. In the study procedures section, this information concerning methods is weak (consent, methods for survey delivery to subjects, average time to complete the survey, what questions were used to assess attitude and practices). Was the survey read to those subjects (if any unable to read?). Who accomplished surveys of the large number of subjects? Were other individuals trained to deploy/record etc? How was the survey invitations delivered to subjects. I think the reader would like to see the survey questions.

Author response: Thank you for providing us with the opportunity to add more information about our study procedures. The Study procedures section has been updated such that it includes the details requested (p4). The survey questions have also been uploaded as supplementary materials (Table S1).

2. The use of the term religious leaders is very broad. How would persons in DR perceive or define what religious leaders mean? This is important since this role was associated with VH- and can the authors hypothesize why? I would think knowing who are the "religious leaders" are would be important for providing implications for decreasing VH.

Author response: Thank you for this feedback. We have now specified in the methods section that religious leaders in the DR relate most commonly to Catholic priests and Protestant pastors (p5, lines 230-231). We have also included an additional paragraph in the discussion to address the potential role of religious leaders in spreading misinformation, as well as suggestions for future research on differential impact by religious denomination (p9, lines 378-386).

Also, a bit of discussion around gender differences in the results would be very interesting.

Author response: Thank you for this suggestion, which gave us the opportunity to include a discussion around gender differences in results. Specifically, we added the following sentence to the discussion section:

“This gender difference has been hypothesized to result from higher exposure among women to vaccine misinformation (e.g., in social media or mother communities), a higher tendency to believe conspiracy theories, as well as lower perceived vulnerability towards COVID-19.[23-25]” (p8, lines 335-338).

Reviewer: 2
Dr. Philip Tarr, University of Basel
Comments to the Author:
Interesting cross-sectional quantitative survey looking into covid-19 vaccine hesitancy (VH) in June-October 2021, after/when covid-19 vaccines became locally available, among a representative sample of household members in the Dominican Republic.

Authors did not apply an established VH instrument, but simply asked participants about intention to obtain covid-19 vaccine. In addition, investigators asked individual, single questions about trust in official (doctors, authorities) and inofficial (social media, religious leaders, traditional media) vaccine information sources.

Overall, covid-19 VH was low (around 5% of the sample), and predictors of VH, even with substantial relative impact (odds ratios >2), had a limited absolute impact on VH.

Authors correctly point out as limitation that people with higher levels of VH may well have declined study participation.

VH towards covid-19 vaccine that was captured in 2021 has limited relevance to today's issues (e.g. has VH increased during the pandemic ? has the pandemic led to increased VH towards established childhood vaccines?)

Also, findings on trust and VH are consistent with previous reports, but the present report is nonetheless welcome because it provides context-specific information related to the Dominican Republic, because of the large sample size and aim to obtain a representative sample.

Author response: Thank you for this overview. We agree that, despite its limitations, this study provides important context on a geographic context that has been the focus of very few research studies. We also hope that the results of the study will have implications beyond 2021, and that it will be relevant for policymakers to understand how trust might shape vaccine acceptance in the context of future epidemics.

Specific comments:

- Figure 1: consider relabeling title and legend as "Vaccine hesitancy SCORE" instead of simply "vaccine hesitancy", because readers may well confuse VH prevalence (in percent) with VH score

Author response: Thank you for this suggestion, which helped increase the clarity and interpretability of Figure 1.

- In the MS title, authors correctly make clear that their study is about VH in relation to covid-19 vaccines, however, this should be made clear throughout the text, i.e. replace all "VH" statements by "covid-19 VH", because study is only about covid-19 vaccine and not about HPV vaccine, pneumococcal, meningococcal, influenza, childhood vaccines etc. In particular, this applies to:
o abstract methods, results, conclusion
o intro (line 33)
o methods (section data classification and analysis)
o results (line 14)
o titles of all tables and figures, including tables S2 and S3

Author response: Thank you again for this feedback, which helps distinguish "traditional" vaccine hesitancy from COVID-19 vaccine hesitancy. We have updated the abstract, introduction (line 141), methods (Data classification and analysis section), results, discussion, tables (Tables 1, 2, S3 [formerly S2], S4 [formerly S3]), and Figure 1 accordingly.

VERSION 2 – REVIEW

**REVIEWER**	O'Malley, Patricia<br>Premier Health System Support- Nursing Research, Nursing Research
**REVIEW RETURNED**	07-May-2024

**GENERAL COMMENTS**	Well written. Text and tables well integrated and organized. Reader will appreciate the authors' results discussion that includes the possible impact of culture and gender.
